# Assessment of health system challenges and opportunities for possible integration of diabetes mellitus and tuberculosis services in South-Eastern Amhara Region, Ethiopia: a qualitative study

**DOI:** 10.1186/s12913-016-1378-6

**Published:** 2016-04-19

**Authors:** Mahteme Haile Workneh, Gunnar Aksel Bjune, Solomon Abebe Yimer

**Affiliations:** Institute of Health and Society, Faculty of Medicine, University of Oslo, Oslo, Norway; Amhara Regional State Health Bureau, Bahir-Dar, Ethiopia; Department of Microbiology, Oslo University Hospital, Oslo, Norway; Department of Bacteriology and Immunology, Norwegian Institute of Public Health, Oslo, Norway

**Keywords:** Diabetes mellitus, Tuberculosis, Challenge, Opportunity, DOTS, Integration, Health system, South-Eastern, Amhara Region, Ethiopia

## Abstract

**Background:**

The double burden of tuberculosis (TB) and diabetes mellitus (DM) is a significant public health problem in low and middle income countries. However, despite the known synergy between the two disease conditions, services for TB and DM have separately been provided. The objective of this study was to explore health system challenges and opportunities for possible integration of DM and TB services.

**Methods:**

This was a descriptive qualitative study which was conducted in South-Eastern Amhara Region, Ethiopia. Study participants included health workers (HWs), program managers and other stakeholders involved in TB and DM prevention and control activities. Purposive sampling was applied to select respondents. In order to capture diversity of opinions among participants, maximum variation sampling strategy was applied in the recruitment of study subjects. Data were collected by conducting four focus group discussions and 12 in-depth interviews. Collected data were transcribed verbatim and were thematically analyzed using NVivo 10 software program.

**Result:**

A total of 44 (12 in-depth interviews and 32 focus group discussion) participants were included in the study. The study participants identified a number of health system challenges and opportunities affecting the integration of TB-DM services. The main themes identified were: 1. Unavailability of system for continuity of DM care. 2. Inadequate knowledge and skills of health workers. 3. Frequent stockouts of DM supplies. 4. Patient’s inability to pay for DM services. 5. Poor DM data management. 6. Less attention given to DM care. 7. Presence of a well-established TB control program up to the community level. 8. High level of interest and readiness among HWs, program managers and leaders at different levels of the health care delivery system.

**Conclusion:**

The study provided insights into potential health systems challenges and opportunities that need to be considered in the integration of TB-DM services. Piloting TB and DM integrated services in selected HFs of the study area is needed to assess feasibility for possible full scale integration of services for the two comorbid conditions.

**Electronic supplementary material:**

The online version of this article (doi:10.1186/s12913-016-1378-6) contains supplementary material, which is available to authorized users.

## Background

The double burden of tuberculosis (TB) and diabetes mellitus (DM) is a significant public health problem in low and middle income countries [1]. There is a large body of evidence demonstrating bidirectional associations between the two diseases. A systematic review showed prevalence rates of TB ranging from 1.7 to 36 % in persons with DM, and DM prevalence ranging from 1.9 to 35 % in patients with TB [2]. In addition to epidemiological associations, the two diseases exacerbate clinical manifestations and affect treatment outcomes of one another [1]. However, despite the known synergy between TB and DM, services for both diseases in most healthcare settings are separately provided. This contributes to missed opportunities for early detection and treatment of both disease conditions [3, 4].

An “integrated management approach” is recommended to deal with the dual burden of DM and TB [4, 5]. Due to the current escalation of DM and its impact on achieving the TB elimination goal, the World Health Organization (WHO) and the International Union against Tuberculosis and Lung Disease (IUATLD) have launched a collaborative framework for care and control of TB-DM comorbidity. The framework focuses on three important intervention strategies namely, establishing mechanisms of collaboration between TB and DM control programs, detection and management of TB in patients with DM, and detection and management of DM in TB patients [6]. China and India have piloted the framework and demonstrated the feasibility of bidirectional screening for both diseases [7–10]. However, more evidence is still required to answer important questions about feasibility of bidirectional screening, optimal ways of delivering treatment, integration of DM and TB services and infection control [4].

Ethiopia is one of the 22 high TB burden countries in the world with an estimated incidence and prevalence rates of 207/100,000 population and 200/100,000 population, respectively [11]. The burden of DM has started to escalate in the country. There were over 1.3 million cases of DM in Ethiopia [12]. In Amhara Region where this study was conducted, the magnitude of TB and DM comorbidity is high [13–15]. The double burden of the two diseases is a serious and growing challenge for the health system in the region. There is no standardized system in place for the provision of integrated services for patients suffering from the dual burden of TB and DM. Integrated services for the two disease conditions may improve early detection, enhances better treatment outcome and help to regularly monitor glycemic control in patients. In this study, we conducted a qualitative study to explore challenges and opportunities of the health system for possible integration of TB and DM services.

## Methods

### Study area and setting

The study was conducted in South-Eastern part of Amhara Region, Ethiopia. South-East Amhara Region consists of four zones and one City Administration namely, North Wollo, South Wollo, Oromia and North Shewa zones, and Dessie City Administration. The total population is estimated at 7,358,301 of whom 3,684,735 were men and 3,673,566 were women. Currently, DOTS is being implemented at all levels of government and a number of private health facilities (HFs). Diagnostic and treatment services for TB patients are provided free of charge. Service for DM is provided in limited number of HFs in the study area and user fee is applicable for DM care [16].

### Study design and data collection

This was a descriptive qualitative study conducted between February and May/2014. The data were collected using focus group discussions (FGDs) and in-depth interview (IDI) techniques. Purposive sampling was applied to select respondents. In order to capture diversity of opinions among participants, maximum variation sampling strategy was applied in the recruitment of study subjects. Participants included health workers (HWs) with varied years of clinical and management experiences. The HWs were selected from public and private HFs located in urban and rural areas. In addition, leaders and program managers working at different levels of the health care delivery system were included as study subjects. Moreover, representatives of Non-Governmental Organizations (NGOs) such as the Ethiopian Diabetic Association and Management Science for Health (HEAL-TB) in Amhara Region were involved as participants of the study. Professional composition of respondents included physicians, health officers, nurses, laboratory technologists, pharmacists, environmental health officers and health extension workers (HEWs).

Open-ended discussion guide was used to facilitate FGDs and IDIs. The discussion guide was composed of questions focusing on health systems challenges and opportunities affecting the provision of TB and DM services, current services provision strategies for TB and DM care, and possibilities of rendering integrated services for the two diseases. The discussion guide was pilot tested before the actual data collection was commenced. Four separate FGDs and 12 IDIs were conducted to collect data. Eight discussants were assigned in each of the FGDs. The IDIs and FGDs continued until saturation was reached. Saturation was reached when no new theme emerged. The principal investigator was responsible for conducting IDIs and moderating FGDs. Minutes were taken by an experienced note taker. All FGDs and IDIs were audio tape recorded. An average of 50 minutes and one and half hour was used to conduct IDIs and FGDs, respectively. The IDIs and FGDs were held in quite private offices which were familiar for the study participants. Various mechanisms were employed to ensure trustworthiness of the study. We selected diversified group of participants representing relevant stakeholders involved in TB and DM services. In addition, we established harmonious relationships and facilitated open and comfortable communications among participants.

### Data analysis

Audio tape recorded data from individual IDIs and FGDs participants were subjected to careful verbatim transcription. The transcribed data were translated from Amharic (local language) into English and were analyzed using QSR International Pty Ltd NVivo 10 software program, 1999–2012. Transcripts of IDIs and FGDs were read and reread by the principal investigator, searching for emergent themes and recurrent ideas. Codes were assigned to segments of the texts, then similar codes were brought together to form a theme. The identified themes were further categorized under the six building blocks of health system as proposed by WHO. The components include health service delivery, health workforce, health management information system (HMIS), medical supplies, health financing and stewardship. These core components were used as analysis framework and in the presentation of study findings [17].

In addition, some quotes that may explain the context of TB and DM services were identified and presented in the respondents own words to give more insight into challenges and opportunities pertaining to integrated TB-DM services.

### Ethical consideration

Ethical approval was secured from the Regional Committee for Research Ethics (REC-Øst), in Norway (reference no. 2013/829/REK sør-øst, dated: 05.06.2013), and the Ethiopian Science and Technology Ministry (reference 3.10/355106 dated: 08/01/06). A written letter of permission from Amhara Regional State Health Bureau, Ethiopia was obtained. Data collection was commenced after receipt of ethical approval. Written informed consent was taken from participants including for audio tape recording. Participant confidentiality was ensured by applying alphanumeric code numbers using cards. The code numbers were used to identify participants during analysis.

## Result

The study participants identified a number of health system challenges and opportunities affecting the integration of TB-DM services. The main themes identified from the analysis were: 1. Unavailability of system for continuity of DM care. 2. Inadequate knowledge and skills of health workers. 3. Frequent stockouts of DM supplies. 4. Patient’s inability to pay for DM services. 5. Poor DM data management. 6. Less attention given to DM care. 7. Presence of a well-established TB control program up to the community level. 8. High level of interest and readiness among HWs, program managers and leaders at different levels of the health care delivery system. Detailed descriptions of the themes are given below right after each of the six WHO health systems building blocks. In addition, illustrative quotes from the data are presented.

## Health service delivery

### Unavailability of system for continuity of DM care

Availability of health care and quality service provision is critical for chronic illnesses management. Almost all participants in this study remarked that DM patients had problems of accessing health services. Limited numbers of HFs in the study area provide DM services with suboptimal quality. This has prevented patients from getting the required services even at times of life-threatening complications. Patients are usually referred to HFs where better services are available.

*“…Unlike TB and HIV in which services are provided at all levels of the health care system in a sustainable manner, DM service is inconsistently rendered mainly in hospitals and limited number of health centers (HCs). For example, there are seven HCs in the district where I am currently working. Out of these, only one HC located at the main town of the district provides limited DM services. Even in this HC, services are frequently interrupted. Whenever patients are suspected or diagnosed as having DM, they are forced to go to the nearby hospital for further investigation and management.”****FGD4-P1***

The clinical care given to DM patients is seriously undermined by lack of coordinated system of care at HFs. All participants described that DM patients are not given priority despite their vulnerability to acute life-threatening complications. Patients in a fasting state queue up for long hours to see their doctor and get their blood tested for FBS in the laboratory. Study participants also noted that there is no separate unit for DM care and patients are managed along with other general health services. Patient follow-up is done based on patients’ own request. In addition, participants stated that there is no established system for patient referral, linkage and feedback mechanism within the health system. This has resulted in lack of continuous medical care and support for DM patients.

“*…Unlike TB and HIV infected patients, there is no established system for DM patients referral and linkage at all levels of HFs. We do not even bother for adherence if a DM patient discontinues treatment and disappears during follow-up periods.”****FGD4-P5***

Contrary to the above stated challenges, the robust TB control program which is decentralized up to primary health care and private HF level was pointed out by all participants as a good entry point for integrating TB-DM services.

*“…TB service in our HC is provided free of charge. It has its own room and assigned HW. Patients are provided with a kit of anti-TB drugs labeled by their own names. They take the tablets in front of a HW. TB suspected patients who arrive at our HC after being referred from health posts by HEWs will not queue up for long hours to get services. The HW working at TB Unit or outpatient department (OPD) will directly send them to the laboratory for sputum smear examination. There is good patient follow-up system. For patients who are unable to come to HFs, HEWs will take the drug and give them at their home. Each TB patient has contact person whose name and telephone number is registered in the TB registration book. This is used for the purpose of tracing patients when they discontinue taking their drug and/or get lost from follow-up. This is a good opportunity.”****FGD3 -P4***

### Perceived workload increase, inadequacy of rooms and risk of TB infection

Participants had mixed feelings regarding perceived workload, room shortage and infection risk during provision of TB-DM integrated services. Some believe that they may be over burdened with work as they will be expected to deliver multiple services. This can affect the quality of their work. They also believe that the existing rooms in the HFs are inadequate and think that the TB-DM integrated services may increase the risk of transmitting TB to DM patients.

*“…Workload can challenge TB-DM integrated services. A number of tasks are expected to be accomplished in rendering integrated services. Due to workload, service quality can be compromised. We may not properly approach the patient and keep records in the correct way. Additional separate room may be required for DM services as that of TB and HIV, otherwise, DM patients can be exposed to TB infection if we give all services in a single room.”****FGD 3-P1***

Other participants did not regard workload, room shortage and risk of TB infection as a problem. As long as available resources are efficiently utilized, modality of integration arranged and infection control measures are established, they think that provision of integrated TBDM service is possible*.*

*“…Workload may not worry us. These days, we are rendering integrated health services. The integration will make health services easily accessible to the patient and tasks become easier to accomplish. Room shortage will not be a problem as far as we are committed. We can rearrange and even start using the existing room. TB already has its own unit. It is just a matter of rearrangement of the existing room for integrated TB-DM services.”****FGD 3- P4***

*“…Transmission of TB infection will not be a problem as long as we provide the service in a separate room using the appropriate referral and linkage system. The same approach is being used for TB and HIV programs. We will take additional infection prevention and control measures.”****FGD 4 -P4***

## Health workforce

### Inadequate knowledge and skills of health workers

Adequate knowledge of HWs about DM management is essential to optimize the quality of care given to DM patients. However, all participants expressed lack of DM knowledge as an obstacle to give integrated TBDM services. They said that there is no ongoing refresher course to update HWs on current knowledge about DM care.

*“…In TB control program, HWs are usually trained and regular refresher trainings are arranged to update them on recent knowledge about TB. Contrary to this, HWs do not get on the job training about DM care. They are dependent on knowledge that was obtained during pre-service training. As a result, DM patients are not properly diagnosed and managed by HWs.”****FGD1-P5***

### Health workers availability

Despite the above mentioned drawbacks, all participants agreed that the presence of a mix of HWs with rich experience in the provision of integrated health service within the existing health system is a good opportunity for the integration of DM into the existing TB control program. They mentioned that the health system of the country through learning by doing had accumulated rich experience in the provision of major health programs using low- and mid-level HWs. This best practice and success story can be adopted to implement integrated TB-DM services.

*“…We don’t have problem of health manpower. We can apply task shifting strategy to increase the number of HWs. In Ethiopia, we have been able to render HIV services by implementing task shifting strategy. For example, for a long time, clinicians and specialist doctors were considered as the only HWs who can provide services for TB and HIV patients. However, by implementing task shifting strategy in recent years, mid-level HWs have been trained in a short time on how to manage TB and HIV patients. This has helped to decentralize and access quality TB and HIV services at peripheral level. We can use a similar approach for TB-DM integrated services. In addition, a large number of mid-level and high-level HW cadres are being trained in the country. Therefore, the problem of trained manpower will not be a problem in the future”****Ind-12***

## HMIS

### Poor DM service data management

In order to make evidence based decision at all level of the health care delivery system, health service programs require quality data register that can be used to assess quality of care, program effectiveness and service utilization. Almost all respondents pointed out that unlike TB control programs, DM service is poorly incorporated in the existing HMIS of the country. There is lack of standardized recording and reporting system for DM services.

*“…DM has never been a discussion agenda during review meetings. It is not planned, monitored and evaluated like TB, HIV and maternal and child health care programs.”****Ind-3***

Respondents on the otherhand stated that TB control program is harmonized in the existing HMIS. It has a well-established recording and reporting system. It also has core indicators to monitor and evaluate the program. Likewise, the existing HMIS being used in the health care delivery system can create a good basis for joint planning, monitoring and evaluation if DM service is integrated with the TB control program.

*“…Currently, HMIS is being implemented at all levels of the health care system in Ethiopia. TB is well incorporated in this system. It has its own registration book at every DOTS unit to register patients and monitor patients’ treatment outcomes. Together with HWs and other stakeholders, we plan, monitor and evaluate TB control program based on data coming from every HF on quarterly basis. This system is a good entry point and can be applied to plan, monitor and evaluate DM services if it is integrated with the TB control program.”****Ind-7***

## Medical supplies

### Frequent stockouts of supplies for DM care

Strong supply management system is fundamental for service quality and sustainability. Stockouts of DM supplies were reported by most of the respondents as one of the chronic problems of HFs. Essential supplies for DM care are intermittently available in HFs. Participants also expressed that they could not get medications and reagents for DM management when they wanted to purchase from wholesale suppliers.

“…*at the HFs level, we do not forecast DM supplies before we run out of stocks. Due to this, we frequently encountered stockouts of DM supplies and were not able to properly manage patients. Basically, this is in contrast to what we normally do to get medical supplies for TB and HIV programs.” FGD4-P4*

Another participant added the following

“…*DM supply is not well incorporated in the current integrated drug supply system which is run by Pharmaceutical Fund and Supply Agency (PFSA). We usually run out of stocks. The required commodities for DM care are not always available even in private wholesale suppliers. Patients are forced to buy drugs from private retail pharmacies with expensive prices*.” ***FGD1-P6***

### Unaffordability of DM supplies

Almost all respondents stated that drugs for DM management are not affordable. Due to frequent stockouts of DM medication in public HFs, patients are obliged to buy costly drugs from private retail pharmacies. Most of the DM patients are poor and do not afford to pay the full cost of the prescribed medications. As a result, patients do not take the full course of medication as prescribed by the doctor. They usually take under dose so that they can use the drug for longer time*.* This affects patient’s treatment adherence and proper control of blood glucose level. One of the participants witnessed the following.

*“…The price of one vial of insulin is more than 75 to 76 birr (about 3.6 USD). One patient may need two vials per month. I usually meet patients who discontinued their medication or took under dose to save the drug for the next month. Most of the time, patients are exposed to high blood glucose level. I know one young boy who died as a result of this action. This all happens due to the high cost of drugs and poor economic conditions of DM patients.”****FGD 3-P3***

Contrary to the above mentioned problems, participants expressed that there is no supply problem for TB services. PFSA periodically delivers TB drugs and reagents to HFs based on the forecast and consumption reports received from HFs. Each TB patient gets the prescribed regimen free of charge.

“…*These days, we don’t have problem of supplies for TB management. PFSA brings us the necessary TB drugs and reagents based on our demand. TB supplies are always available in the HFs. Every TB patient gets anti-TB drug in a kit labeled by his/her name free of charge*.” ***FGD4-P4***

### Well established drug supply management system for TB

Participants acknowledge the current logistic supply chain system as a good opportunity for sustainable medical supply management for both diseases.

*“…We have established integrated drug supply management system for the provision of all essential supplies including TB, malaria and HIV drugs to hospitals and other health institutions. This is run by PFSA. If supplies for DM service are integrated with this system, the required drugs and reagents for both diseases can be available at all times for sustainable provision of services for TB-DM comorbid patients.”****Ind-8***

## Financing

### Patient’s inability to pay for service

All participants mentioned that DM patients have financial constraints and the service is not exempted. This can greatly challenge the integration of DM into the existing DOTS or TB/HIV collaborative services.

“*…User fee is applicable for DM services. Patients are requested to pay out-of -pocket for the service they received. Only children and adults who have poverty certificate are exempted from payment at government health institutions. This imposes a challenge for integration of DM into the existing DOTS or TB/HIV collaborative services”.****FGD 2 -P3***

Participants expressed that DM patients and their families sell their possessions to cover diagnostic and drug costs to the extent that their household resources are depleted. One of the participants shared the following experience.

*“…I know one family whose child is a case of DM for more than 15 years. The child’s father told me that most of the property they possessed had been sold to buy medications. It is only their house which is left now. There is no one who can help them”****Ind-5***

### Availability of health care financing schemes

Respondents noted that the different financing reform schemes that started to operate in the country are opportunities to reduce out-of-pocket expenditure for DM patients in the future. Implementation of community based insurance scheme as a pilot project has been started in few districts.

*“…The start of community health insurance initiatives these days give a chance for the community to get free treatment services when they have health care demands. This can create a good entry point for integrating DM service into the existing TB or HIV services which are provided free of charge.”****Ind-5***

Participants expressed that the current TB and HIV funding opportunity in the existing health service delivery system can be a good opportunity to leverage resource for integrating DM into DOTS or TBHIV collaborative services*.*

*“…TB and HIV services are provided free of charge. The two diseases are covered under the Global Fund supported program. This can be a good entry point for the integration of DM services into the existing TB control program or TB/HIV services.”****FGD 1- P6***

## Stewardship

### Poor attention given to DM care

Respondents pointed out that DM is a neglected disease despite the increasing number of cases from time to time. The attention given for DM care in the existing health care delivery system is marginal. There is no clinical guideline to manage DM patients in a standardized manner. As a result, there is no uniform way of diagnosing and managing DM by HWs.

“…*Because of the low attention given to DM care, there is no guideline or treatment protocol which can be used as a reference. As a result, we have been diagnosing and managing DM patients in haphazard way.”****FGD1-P5***

Contrary to the above, almost all respondents mentioned that there is strong and consistent political commitment to support TB control activities in the existing health care delivery system. Likewise, there was a general consensus among respondents that political leaders and health mangers at all levels would be committed to support DM service if it is integrated with the existing TB control or TB/HIV collaborative program.

*“…These days, TB is the focus of attention for the government as it is one of the Millennium Development Goal (MDG) targets. Different efforts are being exerted to achieve the TB related MDG targets. Integration of DM into the existing DOTS structure can be welcomed by the government as one of the efforts for reducing the TB burden”.****Ind-1***

### Inactive organizational structure for NCDs

Respondents addressed that the existing non-communicable diseases (NCDs) prevention and control structure from federal up to district level is inactive. There is no one assigned at health service delivery point level that is responsible for the overall coordination of NCDs activities in general and DM services, in particular.

*“…Unlike TB, HIV and reproductive health services, DM service does not have a focal person at health institution level. Most of the time, DM services are provided at OPD level by any of the available HW. This negatively affects the quality of management for DM patients.”****FGD 3- P8***

### Poor partner organizations involvement

Resources are always scarce. The government alone cannot meet all the health care needs of patients. Concerted efforts among different actors enhance the provision of integrated health programs. Despite the various DM service gaps present in the existing health care delivery system, there is no partner organization that is currently supporting DM services.

*“…Unlike TB and HIV, there is no partner organization in the existing health care system that supports DM services. There is no one that provides education, material, financial and economic support for DM patients****” FGD3-P2***

Very few participants mentioned that the Ethiopian Diabetic Association is the only partner working for DM control.

*"…The Ethiopian Diabetic Association is an advocate of DM and has 35 branches throughout the country. It provides training for HWs and health education to patients. It is a good partner organization."****Ind-10***

Respondents on the other hand acknowledged that, TB control is partner supported program. Partners are currently doing a commendable job within the existing health service system by providing multi-dimensional support to strengthen the TB control program.

*“…Currently NGOs widely support the existing TB and HIV programs. These can create an opportunity to efficiently utilize resources if DM is integrated into DOTS or TB/HIV collaborative program.”****Ind-1***

Almost all respondents mentioned that the current arrangement made for the organization of community by the government helps to execute all of the desired health service activities. Well-informed and empowered community will be a good partner for successful accomplishment of the integrated program.

*“…These days, urban and rural residents are being organized in small groups to enhance development activities in their communities. Each group has five members. Through this organized system, it is possible to reach every household and health services can effectively be utilized if DM is integrated into the existing DOTS or TB/HIV services.”****Ind-6***

### High level of interest and readiness for TB-DM integration

All participants agreed that provision of TB-DM integrated services within the existing health care delivery system would be possible as long as the gap present in DM service provisions is addressed. They stated that integrated TB-DM service can be given at all levels of public and private HFs by defining specific roles that each HF can play.

*“…It is possible to render TB-DM integrated services at all levels of the existing health institutions including health posts and private facilities. Let alone TB-DM, we can integrate TB/HIV and DM services. At present, it is very common to see many DM and HIV comorbid patients at HFs. The reasons might be drug induced or other factors. We can thus integrate DM into the existing DOTS or TB/HIV collaborative programs, however, the existing different problems affecting the provision of DM services must be addressed in order to provide quality services to patients suffering from the dual burden of TB-DM"****FGD1-P6***

## Discussion

This study explored potential health system challenges and opportunities that need to be considered in the integration of TB-DM services. Even though there are potential challenges, the findings indicate that there is a good opportunity to integrate DM care into the existing TB control program.

Among the challenges identified were poor quality of DM care and absence of monitoring and evaluation systems for NCDs services. Similar findings were observed from previous studies in Gondar, Addis Ababa and Jimma, Ethiopia [18–20]. Other studies in sub-Saharan African countries showed provision of disorganized and unmonitored clinical care for DM patients [21, 22]. Health care systems in many developing countries are mostly designed to address communicable diseases [23–28]. Ensuring availability of health services that meet minimum quality standard is key function of a health system [17]. Currently, services for TB and DM are separately rendered in HFs of the study area. The same is true in many other high TB burden countries. This is a challenge for the provision of quality health service for patients with TB-DM comorbidity. Integrated health service strategy rather than fragmented approach is recommended to ensure continuum of care [29]. Integrated health service is important in contemporary health systems for various reasons. It helps to do more with less resource and is convenient to the people as they receive multiple services at one place or continuum of services through referral [23, 29, 30].

The End TB strategy promotes service integration for the management of TB/HIV and other comorbidities [31]. Integrating DM care into the existing TB control program may increase access and improves quality of care given to TB-DM patients [6, 32, 33]. The internationally recommended DOTS strategy for TB control has been recognized as highly efficient and cost-effective strategy. There is continuous supervision of treatment adherence and patient support in DOTS [34, 35]. This may give a very good opportunity for integrated health education and clinical management of patients suffering from TB-DM comorbidity. Patients diagnosed with DM in the TB units could be linked to DM clinic for confirmation of diagnosis and advices concerning medications, diet and physical exercise. The patient will then continue his/her TB treatment at the TB unit. After the patient has completed the full course of anti-TB treatment, he/she should be sent back to DM clinic for follow-up aiming at detecting TB relapse. The same method could be adopted for DM patients who are diagnosed with TB at the diabetic clinic [4, 5]. This shows that the existing well-functioning TB control program can be adopted to integrate DM care [6, 33].

Some participants seem modest in describing the level of perceived workload during TB-DM service integration. Nonetheless, high workload due to staff shortage, too many data registers activities and increased service uptake by patients could be potential challenges. This will lead to HWs’ lack of time to provide comprehensive and quality services to patients [36]. Adequate staffing through task shifting may be helpful to avert manpower shortage and improve the quality of health service provision to TB-DM patients [23, 26, 28, 29, 37].

Shortage of rooms in HFs can create unfavorable environment to accomplish TB-DM integrated services. Reconfiguration of the existing space or expansion may be needed [6]. This requires a highly committed leadership support from policy makers, program managers and other concerned stakeholders [36].

According to the participants’ opinions, HWs competence related to DM diagnosis and treatment is not up to the standard and this may challenge the integration of TB-DM services [24–28]. In sub-Saharan Africa, HWs lack training on DM patient management [24, 25, 38]. Availability of adequate and trained manpower is essential for the success of integrated health service delivery [29]. Pre-service and on the job trainings of HWs on basic DM care is crucial to provide quality service to TB-DM patients [26, 28, 32]. In addition, the standard TB training given to HWs should continue and incorporate DM management. This will build knowledge, skill and confidence of HWs on the management of patients with the dual burden of TB-DM [28, 36].

The study subjects stated that DM services are not incorporated in the existing HMIS. In most developing countries, there is no established system for monitoring and evaluating NCDs services [21, 22, 39, 40]. A study conducted in Adama, Ethiopia showed lack of separate register, improper data capturing and reporting systems for NCDs [38]. Similarly, findings of pilot studies in China and India showed absence of structured recording and reporting system for monitoring DM services [7, 8]. Establishing a well-developed performance monitoring system is essential to measure the outcome and impact of health service programs [41, 42]. Standardized patient recording and reporting is one of the five components of the DOTS strategy. This important component can be adopted to monitor and evaluate DM services [6, 21, 28, 32–34, 39, 43].

The study participants’ opinions reflect the presence of reliable supply of TB drug and reagents through PFSA. On the otherhand, frequent stockout of DM supplies was stated as a deep rooted problem for DM care [18, 20, 21, 25, 38]. This affects the provision of quality DM care for patients [19, 20]. DM is a lifelong disease and patients need continuous access to proper medical care [24, 26]. Integrated health service requires a continuous supply chain management system [29]. Uninterrupted supply of quality-assured drugs and laboratory supplies is one of the key pillars of the DOTS strategy [32, 34]. Adopting the TB supply chain management system may solve the problem of DM medications stockout in managing diabetic TB patients [23, 32].

Unaffordability of diabetic care costs for TB–DM patients was a serious concern raised by all participants. High cost for DM care is a major constraint for expanding continuity of care for patients [24, 27, 32, 43]. In many countries of the developing world, paying for DM medication is the responsibility of patients [22, 25, 27]. Diabetic patients are usually forced to sell their properties to pay for treatment. The high financial burden and lack of adequate social protection drives patients and their families into poverty [22–24, 26]. In addition, as a result of financial limitations to buy medications, many DM patients do not adhere to the prescribed treatment, and this may lead to poor blood glucose control [24, 25]. Moreover, the current user fee system and unaffordability of DM care costs could be a great obstacle for successful integration of TB and DM services [33]. Social protection on determinants of TB is one of the key components of the End TB strategy [31]. The government may need to make financial risk protection measures either by making the service for DM patients free of charge, or subsidizing high care cost using the different health care financing schemes available in the country [24, 26, 43, 44]. As far as the private for-profit sector is concerned, this solution may be difficult to implement and needs continuous dialogue and agreement. Advocacy work for incorporating DM in the Global Fund financing system as that of TB, HIV and malaria control programs is necessary to generate more funding for DM prevention program [32, 33].

Absence of guideline for DM management was pointed out by respondents as a reflection of poor attention given for DM care. This finding is consistent with the study done in Jimma and Adama, Ethiopia [20, 38]. Many countries in sub-Saharan Africa lack policy framework and guideline for the management of NCDs [24–27, 40]. Lack of guideline can result in variations in the provision of standardized care for DM patients [20]. Integrated health services require supportive policy environment and standardized guideline that operationalizes service delivery and stipulates responsibilities and lines of authorities at various levels of the health system [29, 33, 42]. In this regard, our finding calls for an urgent need of developing standard guideline for DM care.

Contrary to TB control program, under functionality of NCD structure at all levels of the health care delivery system and absence of designated focal persons for DM care at HF level were raised by the study participants as reasons for overlooking DM activities. In many sub-Saharan Africa countries, lack of organizational structures for the management of chronic diseases is a well-known barrier for the execution of desired activities [40]. Making the existing structure active through different capacity building measures and assigning focal persons for NCDs at HF level may strengthens service delivery for NCDs. Political commitment is a corner stone for effective implementation of DOTS and End TB strategy [31, 34]. It is thus crucial that the existing good government commitment and all rounded support given to TB control program are applied for DM care [6].

The finding showed that the numbers of partners that are involved in supporting DM services in the study area are negligible. There are service gaps in the provision of DM care that require partners’ involvement for support. For example, resource mobilization and provision of technical assistance are some of the areas that partner organizations may be involved [32, 45]. It is thus important that NGOs are encouraged to participate in providing the necessary support to ensure access, quality and affordable services for DM patients [24–27].

Engagement of the community through partnership is one of the components of the End TB strategy [31]. Community involvement is one of the key components of the national TB prevention and control strategy in Ethiopia. The community based TB control program in the country has significant contribution in TB suspect identification, referral, and treatment support and follow up of patients [35]. Likewise, integrating DM care into the existing community TB program may improve case finding, patient referral and provision of care and support for people with dual burden of the two disease conditions [26, 28].

The main stakeholders for successful implementation of integrated health services are HWs. Understanding the interest of stakeholders is vital to avoid resistance against integration of health services [23]. Involvement of HWs in decision making can create a sense of ownership and acceptance for change [29]. Therefore, the positive attitude observed among HWs towards the integration of TB and DM services may indicate the acceptance and readiness for rendering integrated TB and DM services in the future.

The strength of this study is that, it is the first study that explored HWs’ opinions on challenges and opportunities of the health system for possible integration of TB and DM services in the study area. The participation of diversified group of health professionals recruited from public and private HFs located in urban and rural areas, the involvement of leaders, program managers and other relevant stakeholders, the reflection of participants’ lived experiences and the high level of agreement among the respondents may increase the validity, reliability and transferability of the study findings.

The study also has limitations. We did not conduct participant observation, document review and quantitative study to support our findings. As a result of this, we may not have fully assessed the natural setting in terms of service provision and program management of both diseases.

### Policy implications

In countries with relatively limited resources for health care, and a rapidly increasing double-disease burden of communicable and NCDs, disease management and control may benefit from an integrated approach by considering ways to take advantage of the existing service provision structure in HFs [28, 36]. Cognizant of this fact, integrating the two diseases benefits to sustain the gain obtained in TB control and may address service gaps in DM care. However, this requires commitment of policy makers to balance the tradeoff between the two diseases by building the health system that can offer financial protection, improved access, and quality services that meet the multiple health needs [23].

## Conclusion

In summary, we identified potential health system challenges and opportunities that should be considered for the integration of DM care into the existing TB control program. Identified challenges affecting DM service provision were unavailability of system for continuity of patient care, risk of TB infection, lack of HWs knowledge, frequent stockouts and unaffordability of supplies for DM care, patient’s inability to pay for service and poor data management. The existing well-functioning TB control program up to the community level and the high level of interest and readiness observed among HWs, program managers and leaders at different level of the health system were identified as good opportunities for integrating TB and DM services. Given the escalating dual burden of TB and DM, and the current service gaps observed in the provision of services for DM patients, there is a need for integrating TB and DM services for providing quality services to patients. However, this may require adequate discussions with relevant stakeholders and decision makers as it has implications for additional resource allocations and other commitments. Piloting TB-DM integrated services in selected HFs of the study area helps to assess feasibility and learn more lessons on the challenges and opportunities of integrated TB-DM services provision.

### Data availability

Discussion guide is attached as an Additional file 1.

## Additional file

Additional file 1: Discussion guide. (DOCX 18 kb)

## Abbreviations

DM: diabetes mellitus
DOTS: directly observed treatment short course
FGD: focus group discussions
HEW: health extension worker
HF: health facilities
HIV: human immuno-deficiency virus
HMIS: health management information system
HW: health workers
IDI: in-depth interview
MDG: Millennium Development Goal
NCD: non –communicable disease
NGO: Non-Governmental Organization
OPD: outpatient department
PFSA: Pharmaceutical Fund and Supply Agency
TB: tuberculosis
TB-DM/HIV: tuberculosis and diabetes mellitus
TB/HIV-DM: tuberculosis and human immuno deficiency virus diabetes mellitus
USD: United State Dollar
WHO: World Health Organization
