# Supplementary material for: Assessment of health system challenges and opportunities for possible integration of diabetes mellitus and tuberculosis services in South-Eastern Amhara Region, Ethiopia: a qualitative study
Source: BMC Health Serv Res. 2016 Apr 19;16:135. doi: 10.1186/s12913-016-1378-6 (PMC4837556; doi:10.1186/s12913-016-1378-6)
Supplement: Additional file 1: — Discussion guide. (DOCX 18 kb) [file 12913_2016_1378_MOESM1_ESM.docx]

**Discussion guide questionnaire**

**Annex -3 English version interview guide questionnaires for /focus group and in-depth interview/ for the study of assessment of health system challenges and opportunities for possible integration of diabetes mellitus and tuberculosis services in South - Eastern Amhara Region, Ethiopia**

**Participants:** Health workers recruited from health post, health center and hospitals, heads of health centers, chief executive officers of hospital , head of districts and Zonal Health Departments, decision makers, experts/ officers at Regional and Federal Ministry of Health, Non-Governmental Organization and Diabetic Association representatives

Participant’s profession: Physician, health officers, nurses, laboratory technologist, pharmacists, environmental health officers and health extension workers.

***Main questions for focus group discussion and in-depth interview***

1**. How is the magnitude of TB, DM in the community?**

**Probe:** how common is it to see TBDM co-morbid patients?

In which community groups are these two diseases common? age , sex, Socio-economic status etc

what relation do you think these two diseases have?

2. **How is the service to TB and DM diseases given in the country?**

**Probe:** is there any bidirectional or integrated service given for TB and DM services in the country? How ?

**3. What are the challenges to provide integrated TBDM services within the existing health care system?**

**Probe:** what problems exist in the existing health care delivery system to give integrated service for both diseases? (system, patient waiting time, perceived work load by health workers , room availability, manpower number and knowledge, health service budget, user fee application , medical supplies availability, affordability and distribution system, HMIS, commitment of leaders, absence of guideline, service demand and utilization by the patient)

**4. What are the opportunities to provide TBDM integrated services within the existing health care system?**

**Probe:** what enabling environments exist in the existing health care delivery system to give integrated service for both diseases? (system, patient

waiting time, perceived work load by health workers , room availability, manpower number and knowledge, health service budget, user fee application, medical supplies availability, affordability and distribution system, HMIS, commitment of leaders, presence of guideline, service demand and utilization by the patient)

**5. Can we give an integrated service for both diseases within the existing health care system?**

**Probe: how/why?**

**6. In which types of health facilities can we give integrated services for both diseases?**

**Probe:** at public or private? why? which level?

**7. Is there something you would like to add?**

**Probe**

**This is the end of our discussion .Thank you very much!!**
